# New approach methodologies to address population variability and susceptibility

**DOI:** 10.1186/s40246-023-00502-7

**Published:** 2023-06-28

**Authors:** Kimberly T. To, Nicole Kleinstreuer, Vasilis Vasiliou, Helena T. Hogberg

**Affiliations:** 1 Inotiv, RTP, Morrisville, NC 27560 USA; 2 NIH/NIEHS/DTT/NICEATM, RTP, Morrisville, NC 27709 USA; 3 Department of Environmental Health Sciences, Yale School of Public Health, New Haven, CT 06520 USA

Human health risk assessment aims to characterize the potential harmful effects of chemical exposures to ensure the safety of broad populations. Among these broad populations are those with higher susceptibility to adverse effects from chemical exposures. Characterizing population-level variability and the interplay of factors that influence heterogeneity of response will help to build a comprehensive understanding of chemical risk that is inclusive, and protective, of susceptible populations. Variation in response to chemicals is determined by a myriad of aspects such as life stage, sex, genomics, epigenomics, nutrition, microbiome, comorbidities, psychosocial stressors, co- and cumulative exposures [1–3]. Genetic variations, such as single nucleotide variants (SNVs), copy number variations (CNVs), or structural variations, can impact how an individual metabolizes and responds to different environmental exposures. Historically, inter-individual differences with respect to potential hazardous effects are addressed by applying default uncertainty factors that include contributions from species extrapolation, sensitive subgroups, toxico-kinetics and -dynamics [4]. However, there are concerns that this may not provide sufficient protection for some populations, and data that inform on chemical-specific adjustment factors would be preferred [5, 6]. Currently, the traditional animal-based toxicology approach is insufficient to inform quantitative assessments of population variability and susceptibility.

Considerable progress has been made in the development and application of new approach methods (NAMs) that are human-relevant and suitable for testing high numbers of chemicals in terms of cost and time. Furthermore, NAMs have the potential to experimentally incorporate variability and susceptibility to derive toxicity predictions that better protect broad populations. Several recent case studies have demonstrated that NAMs can be applied to generate such data informing hazard and risk assessment [7]. For instance, variability of response across multiple donors due to genetics and chronic exposure was demonstrated in a human primary bronchial epithelial cell air–liquid model [8]. Genetic variability and environmental exposures have also been evaluated using cell lines [9], induced pluripotent stem cells (iPSCs) [10, 11], in silico models [12], and small model organisms, e.g., Zebrafish [13], Elegans [14] and Drosophila [15]. NAMs have been applied to assess additional factors that contribute to variability and susceptibility such as sex [16, 17], life stage [16, 18, 19], and comorbidities [20], including rare diseases [21]. Moreover, NAMs have the potential to incorporate complex mixtures and cumulative exposures [22–24]. Probabilistic methods can incorporate variability into predictions and have been used to derive reference dose estimates [25] and points of departure [23]. Understanding how variability and susceptibility factors are associated with exposure responses will help to identify susceptible populations and support NAMs-based risk assessment paradigms by quantifying and controlling for known sources of variability [3].

Certain populations who are more susceptible to chemical exposures encounter health disparities resulting from various factors that may include cumulative impacts, psychosocial stressors, or complex mixtures, among others. NAMs have the potential to elucidate the mechanisms underlying long-term exposure; however, community engagement is imperative to conducting meaningful, impactful research. In this collection, we welcome cutting-edge research on developing, applying, and validating NAMs that are designed to represent population variability and susceptibility and ensure better human health protection for the most vulnerable and sensitive individuals among us.
